# Acknowledgement to Reviewers of *Membranes* in 2013

**DOI:** 10.3390/membranes4010079

**Published:** 2014-02-24

**Authors:** Membranes Editorial Office

**Affiliations:** MDPI AG, Klybeckstrasse 64, CH-4057 Basel, Switzerland

The editors of *Membranes* would like to express their sincere gratitude to the following reviewers for assessing manuscripts in 2013:

Aili, David
Albertin, Luca
Barredo-Damas, S.
Basile, Angelo
Bellona, Christopher
Besser, Ronald S.
Borsi, Iacopo
Bracewell, Daniel G.
Bredesen, Rune
Budd, Peter
Cazorla-Amorós, Diego
Chang, Feng-Chih
Chen, Jinhua
Cheng, Liao-Ping
Cho, Jinwoo
Chu, Benjamin
Chu, Peter Po-Jen
Chung, Tai-Shung
Ciardelli, Francesco
Cohen, Yoram
Coronas, Joaquín
Deeke, Alexandra
Deng, Baolin
Escobar, Isabel C.
Fong, Hao
Fullerton-Shirey, Susan K.
Gallucci, Fausto
Giannici, Francesco
Gilron, Jack L.
Granville, Anthony M.
Groth, Andrew
Hägg, May-Britt
Herzing, Andrew
Hesampour, Mehrdad
Hoek, Eric M. V.
Hong, Liang
Janiak, Christoph
Karabelas, A. J.
Kern, Wolfgang
Khatib, Sheima Jatib
Kim, In-Chul
Kim, Jeonghwan
Kuznicki, Steven M.
Kyu, Thein
Lawler, Jenny
Lee, Kew-Ho
Liné, Alain
Linkous, Clovis A.
Lu, Xiaoyun
Luis, Patricia
Ma, Guang-Hui
Malaisamy, Ramamoorthy
Maranas, Janna K.
Marcos, Bernard
Mather, Glenn C.
Mazzei, Rosalinda
Menkhaus, Todd J.
Miyoshi, Shogo
Moore, Robert B.
Naessens, Wouter
Nikkola, Juha
Nopens, Ingmar
Nørgaard, C. F.
Ortiz, Inmaculada
Pant, Deepak
Paraskeva, Christakis A.
Park, Sang-Eon
Ramon, Guy
Rodrigues, Alírio E.
Romanos, George E.
Rustad, James R.
Samhaber, W. M.
Schiffman, Jessica D.
Schröder, Uwe
Serra, Jose M.
Servili, Maurizio
Sleutels, Tom H. J. A.
Smart, Simon
Teli, Shivanand B.
Tseng, Hui-Hsin
Van Rijn, Cees Jm
Winnubst, Louis
Wu, Rome-Ming
Yamaki, Junichi
Yamaki, Tetsuya
Yampolskii, Yuri
Zhang, Liangmin

